# Effects of boxing exercise in people with Parkinson’s disease: a systematic review

**DOI:** 10.3389/fnagi.2025.1505326

**Published:** 2025-03-27

**Authors:** Zhihai Wang, Baofu Song, Cong Liu, Huihui Ma, Zirong Bai, Marcelo A. S. Carneiro, Layale Youssef, Chao Chen, Lingli Zhang, Dan Wang, Dexin Wang

**Affiliations:** ^1^School of Athletic Performance, Shanghai University of Sport, Shanghai, China; ^2^Liaocheng Infant Normal School, Liaocheng, China; ^3^School of Physical Education, Shanghai University of Sport, Shanghai, China; ^4^Surgical Outcomes Research Centre (SOuRCe), Royal Prince Alfred Hospital (RPAH), Sydney, NSW, Australia; ^5^Faculty of Medicine and Health, Central Clinical School, The University of Sydney, Sydney, NSW, Australia; ^6^Metabolism, Nutrition and Exercise Laboratory, Physical Education and Sport Center, Londrina State University, Londrina, Brazil; ^7^École de Kinésiologie et des Sciences de l’Activité Physique, Université de Montréal, Montreal, QC, Canada; ^8^School of Physical Education, Dalian University, Dalian, China

**Keywords:** Parkinson’s disease, boxing, exercise, movement disorder, rehabilitation, physiotherapy

## Abstract

**Objective:**

Parkinson’s disease (PD) is a chronic neurodegenerative disorder characterized by progressive changes in both motor and non-motor symptoms. Boxing exercise can improve PD symptoms. This review aimed to determine the effects of boxing exercise on lower extremity strength, balance, mobility, gait, depression, quality of life, disease severity, exercise safety, and adherence in patients with PD.

**Design:**

A systematic review.

**Setting and participants:**

Articles were selected if they included participants diagnosed with PD and used boxing exercise as the main intervention.

**Methods:**

Systematic review study based on PRISMA criteria. Searches were implemented in PubMed, EMBASE, Web of Science, and Cochrane Library until February 2024. We selected studies reporting on the pre-post assessment of a boxing intervention with lower extremity strength, balance, mobility, gait, depression, quality of life, disease severity, exercise safety, and adherence in patients with PD. Two independent reviewers conducted study selection, data extraction, and quality assessment. The Physiotherapy Evidence Database and ROBINS-I 2.0 criteria evaluated the literature’s quality.

**Results:**

Out of 4,301 records, 13 studies were included, involving 402 PD patients aged 53–89, with 72.4% being male. Interventions lasted 6–96 weeks, primarily in community settings and gymnasiums. Moderate-quality evidence suggested boxing exercises is feasible and effective for enhancing lower extremity strength, balance, mobility, gait, depression, quality of life, disease severity, exercise safety and adherence in PD patients.

**Conclusions and implications:**

Boxing exercise can effectively improve both motor and non-motor symptoms in PD patients, with safety and high adherence. This review systematically summarizes the emerging evidence on the application of boxing exercise in the rehabilitation of patients with PD. Future research should include more homogenous PD patient populations and conducting randomized controlled trials.

## 1 Introduction

Parkinson’s disease (PD) is a prevalent neurodegenerative disorder, recognized as the second most common worldwide (Chou et al., 2017). It is estimated that 10 million individuals worldwide are affected by PD, with a prevalence rate of 1 to 2 per 1,000 individuals which increases with age (Ou et al., 2021). Thus, the number of people with PD is expected to exceed 12 million by 2040 (Dorsey and Bloem, 2018). PD is characterized primarily by a triad symptom: rigidity, tremor, and bradykinesia (Bloem et al., 2021). It includes motor symptoms such as hypokinesia, rigidity, tremor, and postural instability, alongside non-motor symptoms including depression, anxiety, fatigue, and sleep disturbances, which are frequently overlooked (Kalia and Lang, 2015). These multiple symptoms can lead to functional limitations, such as reduced mobility, impairment in gait speed, increasing the risk of falls, fractures and brain injuries. This significantly impacts the quality of life, increases premature mortality, and hence imposes a substantial burden on families and society (Song et al., 2017). Given these implications, it is crucial to identify and implement effective intervention strategies to alleviate symptoms and slow the progression of PD in affected patients.

PD is currently considered incurable, with pharmacotherapy serving as the primary treatment approach (Fox et al., 2018). However, the high cost of medications and their decreasing efficacy over time, and adverse side effects such as orthostatic hypotension, somnolence, and hallucinations significantly impair health and muscle function (Fahn, 2018). Furthermore, pharmacologic interventions are also insufficient to addressing non-motor impairments of PD (Pedersen et al., 2012). Recent evidence has strongly suggested that exercise can effectively complement pharmacotherapy, particularly high-intensity exercise, which may slow PD progression by promoting neuroplasticity (Frazzitta et al., 2014; Tollár et al., 2018). In this regard, boxing exercise (a type of high-intensity exercise) has become increasingly popular among patients with PD (Blacker et al., 2023; Combs et al., 2013; Patel et al., 2023; Sangarapillai et al., 2021). It is estimated that over 4,500 patients with PD globally are currently participating in boxing programs (Morris et al., 2019). Current studies have shown that boxing exercise can effectively improve gait function (Combs et al., 2013; Sangarapillai et al., 2021; Shearin et al., 2021), mobility (Combs et al., 2013; Combs et al., 2011; Domingos et al., 2022), and balance (Combs et al., 2013; Domingos et al., 2022; Sonne et al., 2021) in individuals with PD. Additionally, boxing has been found to reduce depressive symptoms (Patel et al., 2023), alleviate disease severity (Patel et al., 2023; Sangarapillai et al., 2021), and enhance quality of life (Combs et al., 2013; Combs et al., 2011; Sangarapillai et al., 2021). Notably, patients have demonstrated high adherence to boxing interventions without experiencing falls or other adverse events (Blacker et al., 2023; Sangarapillai et al., 2021). Boxing exercise is a sport that integrates speed, strength, and endurance (Dawson et al., 2020; Sonne et al., 2021). For instance, boxing exercise requires rapid lower limb movements in various directions and planes (Shearin et al., 2021). It also involves upper limb punching which requires quick arm movements, trunk rotation, and postural adjustments (Combs et al., 2011). Compared to other forms of exercise, such as aerobic and resistance training, boxing offers a comprehensive range of activities, including aerobic, strength, stretching, balance, and agility exercises, involving a wide spectrum of movement benefits (Patel et al., 2023). Nonetheless, ensuring adherence to an exercise program and the safety profile of the program itself remain crucial concerns for participants (Morris et al., 2019; Rhodes et al., 1999). To the authors’ knowledge, only one systematic review (Morris et al., 2019) has reviewed the evidence on the benefits and risks of boxing exercises for PD patients. This systematic review (Morris et al., 2019) included just one small randomized controlled trial (Combs et al., 2013) and one case series evaluation (Combs et al., 2011). The results indicate that the benefits, precautions, contraindications, and limitations of boxing exercises for PD patients remain verified.

Considering the increasing burden of PD with patients and the potential of boxing exercise as an intervention, there is an urgent need to systematically explore the effects of this exercise. To our knowledge, there is a lack of latest systematic reviews to addressed this gap. Building on the above considerations, the primary aim of the present study was to evaluate the efficacy of boxing exercises on various aspects including motor symptoms (lower extremity strength, balance, mobility, and gait), non-motor symptoms (depression, quality of life, and disease severity), exercise safety and adherence individuals with PD. In addition, this systematic review aims to identify research gaps and propose suggestions for future studies, thereby providing robust evidence-based recommendations for clinical practice.

## 2 Methods

This systematic review was performed in compliance with the PRISMA guidelines (Page et al., 2021) and has been registered in the PROSPERO database (CRD42024518202).

### 2.1 Search strategy

We retrieved literature published in the English language from the inception of the databases up to February 2024, by accessing PubMed, Web of Science, EMBASE, and Cochrane Library. Our search strategy incorporated a combination of MeSH terms and free-text terms, with the specific strategies for each database detailed in Table 1. Furthermore, we evaluated existing systematic reviews and the reference lists of the included studies to supplement our search for the relevant literature. In addition, after the exclusion of duplicate publications, the gray literature was excluded, including conference abstracts and/or papers, thesis and/or dissertations, meta-analyses, systematic reviews, commentaries, and editorials. In case of disagreements, a third reviewer (HM) evaluated the article.

**Table 1:** The database search strategy.

Database	Retrieval strategy
Cochrane Library and PubMed	#1 “Boxing”[Mesh] OR “Boxing exercise” [Title/Abstract] OR “Box”[Title/Abstract] OR “Boxercise” [Title/Abstract] OR “Combat” [Title/Abstract]
	#2 “Parkinson Disease, Secondary”[Mesh] OR “Paralysis Agitans”[Title/Abstract] OR “Primary Parkinsonism” [Title/Abstract] OR “Parkinson’s Disease” [Title/Abstract] OR “Secondary Parkinsonism” [Title/Abstract] OR “PD” [Title/Abstract] OR “Symptomatic Parkinson Disease” [Title/Abstract] OR “Idiopathic Parkinson Disease” [Title/Abstract] OR “Parkinsonism, Primary” [Title/Abstract] OR “Secondary Parkinson Disease” [Title/Abstract]
	#3 #1 AND #2
EMBASE	#1 “Parkinson Disorders”[exp]
	#2 “Parkinson Disease, Secondary”[ab,ti] OR “Paralysis Agitans”[ab,ti] OR “Primary Parkinsonism” [ab,ti] OR “Parkinson’s Disease” [ab,ti] OR “Secondary Parkinsonism” [ab,ti] OR “PD” [ab,ti] OR “Symptomatic Parkinson Disease” [ab,ti] OR “Idiopathic Parkinson Disease” [ab,ti] OR “Parkinsonism, Primary” [ab,ti] OR “Secondary Parkinson Disease” [ab,ti]
	#3 #1 OR #2
	#4 “Boxing”[exp]
	#5 “Boxing exercise” [ab,ti] OR “Box” [ab,ti] OR “Boxercise” [ab,ti] OR “Combat” [ab,ti]
	#6 #4 OR #5
	#7 #3 AND #6
Web of Science	#1 TS = (“Boxing” OR “Boxing exercise” OR “Box” OR “Boxercise” OR “Combat”)
	#2 TS = (“Parkinson Disease, Secondary” OR “Paralysis Agitans” OR “Primary Parkinsonism” OR “Primary Parkinsonism” OR “Secondary Parkinsonism” OR “Symptomatic Parkinson Disease” OR “Idiopathic Parkinson Disease” OR “Parkinsonism, Primary” OR “Secondary Parkinson Disease”)
	#3 #1 AND #2

### 2.2 Eligibility criteria

Two independent researchers (ZW and CL) conducted a systematic search and selection of literature, consulting a third researcher (HM) to reach a consensus in cases of disagreement.

The eligibility criteria were as follows: (1) adults diagnosed with PD; (2) interventions featuring boxing exercises, either solo or combined with other forms of exercise or physiotherapy; (3) outcomes included strength, balance, mobility, gait, depression, quality of life, disease severity, safety and adherence; (4) study designs including randomized controlled trials (RCTs), controlled clinical trials, cohort studies, case series, case–control studies, and quasi-experimental studies; (5) original articles peer-reviewed without limitations on publication date, published until February 2024; (6) published in English language.

The exclusion criteria were as follows: (1) studies involving participants with stroke, traumatic brain injury, and/or non-motor neurological diseases; (2) interventions other than boxing; (3) unavailable full texts; (4) review articles (including meta-analyses and systematic reviews), conference abstracts, dissertations, and commentaries; (5) duplicate publications.

### 2.3 Study selection

Upon identifying eligible studies from each database, the retrieved literature was first imported into EndNote X9 (Milne-Ives et al., 2020) to consolidate and eliminate duplicates. Two researchers (ZW and CL) independently screened the titles and abstracts to identify potentially relevant articles. A full-text review was then performed for further selection of the initially selected literature. When data were incomplete or unextractable, the authors were contacted to provide the missing information. The extracted content included essential details such as authors, publication year, country, and study design; participant baseline information including sample size, gender, mean age of participants, and mean Hoehn and Yahr score; intervention details like type, specifics, regimen, duration, or dosage; and the main findings.

### 2.4 Methodological quality assessment

The methodological quality of the included literature was independently assessed by two researchers (ZW and CL), with any disagreements resolved through discussion with a third researcher (HM) to reach a consensus. The Physiotherapy Evidence Database (PEDro) was used to evaluate the quality of RCTs, which consisted of 11 evaluation criteria. Each criterion met was given one point, with a maximum score of 10. The first evaluation criteria did not contribute to the final score. According to the scale, scores are categorized as follows: <4 indicates poor quality, 4–5 fair quality, 6–8 good quality, and 9–10 high quality (Fernández-Landa et al., 2023). For non-randomized controlled trials (non-RCTs), the ROBINS-I 2.0 criteria were used, which consisted of seven items that primarily assessed the risk of bias as low, moderate, serious, critical, or lacking information (Sterne et al., 2016).

## 3 Results

### 3.1 Search strategy and criteria

**Figure 1:**
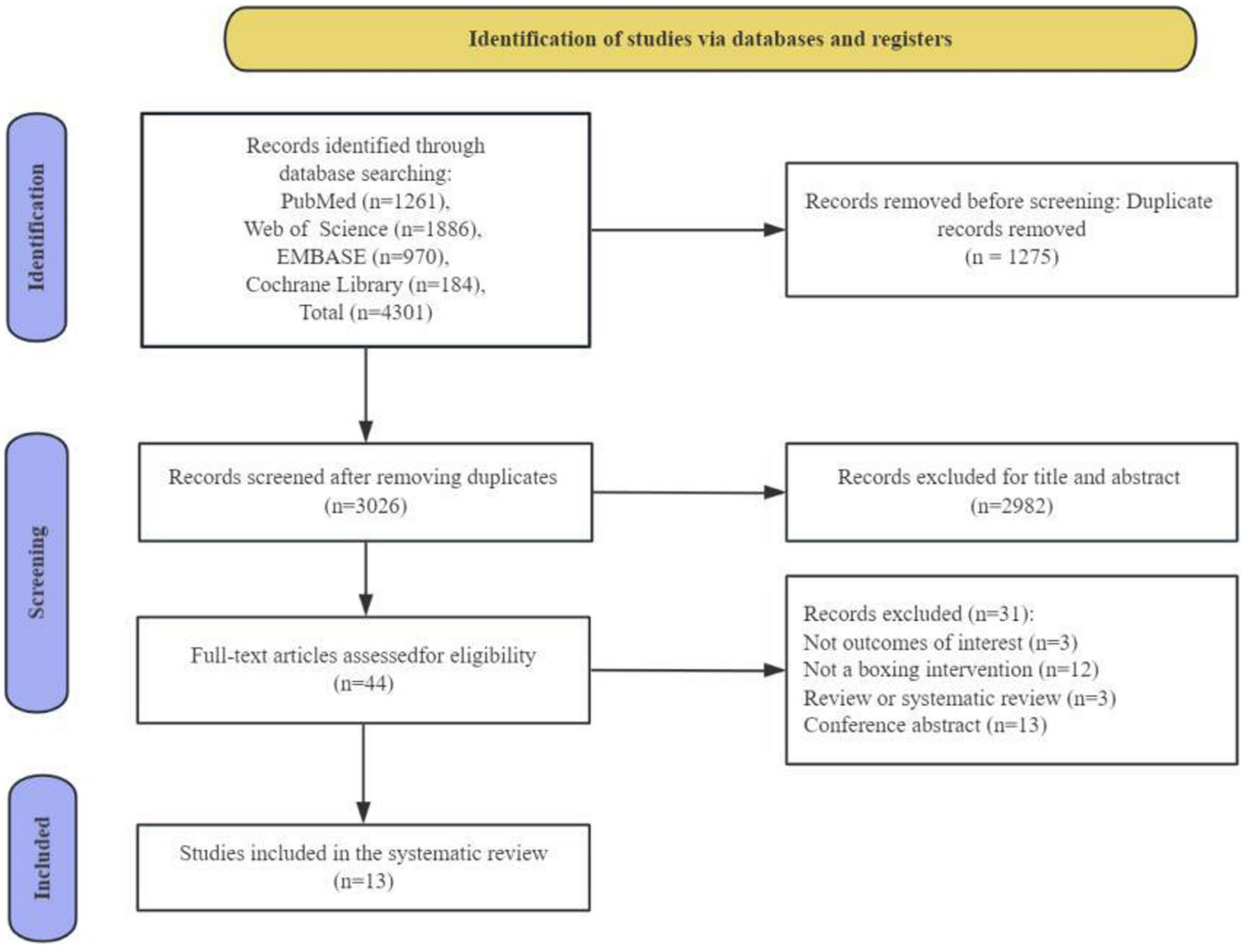
Flow diagram of the systematic review for inclusion/exclusion of studies.

A systematic search yielded 4,301 records as documented in the PRISMA flow diagram (Figure 1). After removing duplicates and screening the gray literature, including conference abstracts and/or papers, thesis and/or dissertations, meta-analyses, systematic reviews, commentaries, and editorials, 44 articles were preliminarily selected according to eligibility criteria. Following a thorough full-text evaluation, 31 articles were excluded for not meeting the inclusion criteria due to irrelevant outcomes (*n* = 3), not a boxing intervention (*n* = 12), review or systematic review article (*n* = 3), and conference abstract (*n* = 13). Therefore, the final analysis included 13 studies (Blacker et al., 2023; Combs et al., 2013; Combs et al., 2011; Dawson et al., 2020; Domingos et al., 2022; Hermanns et al., 2021; Horbinski et al., 2021; Moore et al., 2021; Patel et al., 2023; Sangarapillai et al., 2021; Shearin et al., 2021; Sonne et al., 2021; Urrutia et al., 2020).

### 3.2 Methodological quality

The quality assessment results are detailed in Tables 2, 3. Using the PEDro scale, three RCTs were evaluated, indicating a low risk of bias with an average score of 8.33. This suggests that the results of the systematic review are trustworthy. Notably, one study achieved the maximum score of 10. The absence of blinding for therapists and participants in two studies may be due to inherent experimental design constraints, presenting significant challenges to blinding and potentially impacting the methodological integrity of the included studies. The ROBINS-I 2.0 tool was used to assess the quality of ten non-RCTs studies, indicating an overall low risk of bias. Nevertheless, given that all included studies involved exercise interventions with human participants, there was an increased risk of bias due to deviations from the intended interventions.

**Table 2:** Randomized controlled trial (PEDro scale).

Study	Criteria											Score	Result
	1	2	3	4	5	6	7	8	9	10	11		
Combs et al. (2013)	Y	Y	N	Y	N	N	Y	Y	Y	Y	Y	7	Good
Sangarapillai et al. (2021)	Y	Y	Y	Y	Y	Y	Y	Y	Y	Y	Y	10	Excellent
Domingos et al. (2022)	Y	Y	Y	Y	N	N	Y	Y	Y	Y	Y	8	Good

N: Did not meet the criteria; Y: Met the criteria. 1. Eligibility criteria were specified; 2. Random allocation; 3. Concealed allocation; 4. Baseline similar; 5. Blinded participant; 6. Blinding of all therapists; 7. Blinding of all assessors; 8. Adequate follow-up; 9. Intention to treat analysis; 10. Between group analysis; 11. Outcome measure data point estimates and variability.

**Table 3:** Non-randomized controlled trial (ROBINS-I 2.0).

First author, year	1	2	3	4	5	6	7
Patel et al. (2023)	Low	Low	Low	Serious	Low	Low	Low
Hermanns et al. (2021)	Low	Low	Low	Serious	Low	Low	Low
Blacker et al. (2023)	Low	Low	Low	Serious	Moderate	Low	Low
Urrutia et al. (2020)	Low	Low	Low	Serious	Moderate	Low	Low
Shearin et al. (2021)	Low	Low	Low	Serious	Moderate	Low	Low
Combs et al. (2011)	Low	Low	Low	Serious	Low	Low	Low
Moore et al. (2021)	Low	Low	Low	Serious	Serious	Low	Low
Horbinski et al. (2021)	Moderate	Low	Low	Serious	Serious	Low	Low
Sonne et al. (2021)	Moderate	Low	Low	Serious	Serious	Low	Low
Dawson et al. (2020)	Moderate	Low	Low	Serious	Moderate	Low	Low

1: Confounding; 2: Selection bias; 3: Bias in measurement classification of interventions; 4: Bias due to deviations from intended interventions; 5: Bias due to missing data; 6: Bias in measurement of outcomes; 7: Bias in selection of the reported result.

### 3.3 Study and sample characteristics

The analysis covered 13 publications (Blacker et al., 2023; Combs et al., 2013; Combs et al., 2011; Dawson et al., 2020; Domingos et al., 2022; Hermanns et al., 2021; Horbinski et al., 2021; Moore et al., 2021; Patel et al., 2023; Sangarapillai et al., 2021; Shearin et al., 2021; Sonne et al., 2021; Urrutia et al., 2020) from four countries: the United States, Australia, Canada, and the Netherlands. Notably, 10 of these studies (76.9%) were conducted in the United States (Combs et al., 2013; Combs et al., 2011; Dawson et al., 2020; Hermanns et al., 2021; Horbinski et al., 2021; Moore et al., 2021; Patel et al., 2023; Shearin et al., 2021; Sonne et al., 2021; Urrutia et al., 2020), with the majority published between 2020 and 2023 (84.6%) (Blacker et al., 2023; Dawson et al., 2020; Domingos et al., 2022; Hermanns et al., 2021; Horbinski et al., 2021; Moore et al., 2021; Patel et al., 2023; Sangarapillai et al., 2021; Shearin et al., 2021; Sonne et al., 2021; Urrutia et al., 2020). The study designs included three RCTs (Combs et al., 2013; Dawson et al., 2020; Domingos et al., 2022) and ten non-RCTs (Blacker et al., 2023; Combs et al., 2011; Dawson et al., 2020; Hermanns et al., 2021; Horbinski et al., 2021; Moore et al., 2021; Patel et al., 2023; Shearin et al., 2021; Sonne et al., 2021; Urrutia et al., 2020). All participants were adult patients with a confirmed diagnosis of PD, totaling 402 patients. Patient ages ranged from 40 to 90 years, with a male gender bias (72.4% male). The average disease duration and Hoehn and Yahr scores varied from 2.4 to 7.9 years and stages 1–4, respectively. Three studies omitted details on disease severity (Dawson et al., 2020; Domingos et al., 2022; Horbinski et al., 2021), one failed to specify the gender distribution of its participants (Sangarapillai et al., 2021), and one study exclusively examined male participants (Combs et al., 2011). Detailed information for each included study is provided in Table 4.

**Table 4:** Characteristic of studies included (*n* = 13).

Study, year, nation	Duration (week)	Study design	Sample size (M, F), Mean age (year)	Mean Hoehn and Yahr	Disease duration (year)	Medications
Patel et al. (2023) United States	12	Non-RCTs	14 (M:8, F:6), 62.2	2	7.9 (4.4)	Dopaminergic medication uses in 12 participants; Deep brain stimulation uses in 2 participants
Blacker et al. (2023) Australia	15	Non-RCTs	10 (M:6, F:4), 60	1–2	4.7 (3)	Pramipexole, Stalevo Rotigone Madopar Rasagaline Safinamide
Sangarapillai et al. (2021) Canada	10 (11–20 follow-up)	RCTs	40 (NR), 64.4	2.5	EG: 6.38 (4.9); SG: 7.82 (5.2)	Levodopa equivalent therapy
Combs et al. (2013) United States	12	RCTs	31 (M:21, F:10), 67.3	2	TG: 4.1 (8.3); EG: 3.5 (15.2)	NR
Urrutia et al. (2020) United States	6 (7–12 follow-up)	Non-RCTs	15 (M:10, F:5), 66.1	1–3	5.5 (4.08)	NR
Shearin et al. (2021) United States	12	Non-RCTs	26 (M:20, F:6), 68.4	1–3	4.75	NR
Combs et al. (2011) United States	12	Non-RCTs	6 (M:6, F:0), 60.2	1–4	28.67 (24.34)	Dopamine replacement therapy in 4 participants
Dawson et al. (2020) United States	16	Non-RCTs	47 (M:34, F:13), 68.3	NR	4.24 (4.55)	NR
Hermanns et al. (2021) United States	12	Non-RCTs	6 (M:4, F:2), 40–90	1–3	0–16+	NR
Moore et al. (2021) United States	24	Non-RCTs	12 (M:9, F:3), 67.0	1–3	NR	NR
Horbinski et al. (2021) United States	64	Non-RCTs	98 (M:76, F:22), 70.6	NR	NR	NR
Domingos et al. (2022) Netherlands	10	RCTs	29 (M:14, F:15), 64	NR	5.5 (4.3)	Dopamine medication uses in 23 participants
Sonne et al. (2021) United States	96	Non-RCTs	68 (M:54, F:14), 71.2	1–3	NR	NR

RCTs, randomized controlled trials; non-RCTs, non-randomized controlled trials; M, male; F, female; NR, no reported; EG, boxing group; SG, sensor group; TG, traditional exercise group.

### 3.4 Interventions conducted and dosing

The boxing exercise program primarily employed a circuit training approach, incorporating boxing drills, resistance training, agility exercises, and endurance training (Blacker et al., 2023; Combs et al., 2011; Dawson et al., 2020; Song et al., 2017). Participants engaged in these activities using gloves and punching bags, without any physical contact with others. The interventions were mainly conducted in community (Combs et al., 2013; Combs et al., 2011; Domingos et al., 2022; Moore et al., 2021; Patel et al., 2023; Shearin et al., 2021; Urrutia et al., 2020) and gymnasiums (Dawson et al., 2020; Hermanns et al., 2021), although four studies did not specify the locations (Blacker et al., 2023; Horbinski et al., 2021; Sangarapillai et al., 2021; Sonne et al., 2021). The duration of the interventions varied from 6 to 96 weeks, with sessions occurring 1–3 times per week and lasting an average of 60–90 min. Sample sizes ranged from a minimum of 6 participants (Combs et al., 2011; Hermanns et al., 2021) to a maximum of 68 participants (Sonne et al., 2021). In terms of intensity, three studies implemented high-intensity programs (Moore et al., 2021; Sangarapillai et al., 2021; Urrutia et al., 2020), two studies instructed participants to complete the entire program at maximal intensity (Combs et al., 2013; Combs et al., 2011), one study used moderate intensity (Shearin et al., 2021), another progressed from low to high intensity (Blacker et al., 2023), and one relied on coach-directed intensity without standardization (Patel et al., 2023). Five studies did not report the exercise intensity (Dawson et al., 2020; Domingos et al., 2022; Hermanns et al., 2021; Horbinski et al., 2021; Sonne et al., 2021). The primary outcome measures included lower extremity strength (15 s and 30 s sit-to-stand; 30 s Chair Stand, 30 s CST), balance (Fullerton Advanced Balance scale, FAB; Berg Balance Scale, BBS; Mini-Balance Evaluation Systems Test, Mini-BESTest; ABC, Activity Specific Balance Confidence Scale; 30 s single leg stance), mobility (Timed up and go, TUG; dual-task TUG, dTUG), gait (10 Meter Walk Test, 10MWT; 6 min walk test, 6MWT; 6 min walk distance, 6MWD), depression (Center for epidemiologic studies depression, CES-D; Hamilton Depression Rating Scale, HDRS), quality of life (Parkinson’s Disease Questionaire-39, PDQ-39; Parkinson’s Disease Quality of Life scale, PDQL; Brief quality-of-life survey the Euroquol-5D, EQ-5D), and disease severity (Unified Parkinson Disease Rating Scale, UPDRS; Movement Disorders Society Unified Parkinson’s Disease Rating Scale part III, MDSUPDRS III). These measures aimed to comprehensively evaluate the effects of boxing exercises on both motor and non-motor functions in patients with PD. Table 5 provides detailed information on the specific outcome measures and main results.

**Table 5:** Characteristic of interventions and results of studies included (*n* = 13).

Study (year)	Intervention	Intervention parameters	Intensity	Intervention location	Adherence	Outcome measure	Main results
Patel et al. (2023)	*Boxing:* 30-min warm up exercises and socialize (arrive 30 min early) 60-min boxing training, including footwork, shifting, strikes/punches, combination strikes and Jump rope techniques. Finally, incorporated mental performance concepts, including breathing techniques, visualization and mental rehearsal, goal setting and self-talk	60 min, 2×/week, 12 weeks	The intensity of exercise in each class was not specifically standardized, and varied per the discretion of the instructor	Community	80%	HDRS, PDQ-39, MDSUPDRS III	Depression↑ Quality of life↑ Disease severity↑
Blacker et al. (2023)	*Boxing:* 10-min warm up exercises 35-min boxing circuit training, including differing boxing stances and movements (10 min), unidirectional and bidirectional punches (25 min) 15-min cool down exercises	60 min, 3×/week, 15 weeks	Block 1: low to moderate intensity Block 2: high intensity interval training Block 3: 70–80% APMHR, RPE to 13–15	NR	100%	MDSUPDRS III	Disease severity↑
Sangarapillai et al. (2021)	*RSB:* Consisted of a warm-up, boxing specific exercise (high-intensity boxing drills, shadow boxing, jumping jacks, and speedbag drills) and a cool down *Sensory exercise:* Consisted of a warm-up, sensory specific exercise (stretches, walking, and chair exercises where participants were encouraged to complete the exercises slowly, in a controlled manner and with their eyes closed) and a cooldown	60 min, 3×/week, 10 weeks (11–20 follow-up)	High intensity	NR	98%	Stride length, gait velocity, PDQ-39, UPDRS	Stride length↓ Gait velocity↓ Quality of life↑ Disease severity↓
Combs et al. (2013)	*Boxing:* 15-min warm up exercises (seated) 45–60 min boxing circuit training, including functional, endurance, and punching exercises *Traditional exercise:* 15-min warm up exercises (seated) Strengthening, endurance, and balance exercises 3. 15-min cool down exercises	60 min, 2–3×/week, 12 weeks	Encouraging participants to train as intensely as they could tolerate and by striving to complete more repetitions during each training	Community	71%	BBS, ABC, TUG, dTUG, Gait velocity, 6MWT, PDQL	Balance↑ Balance confidence↑ Mobility↑ Gait velocity↑ Gait endurance↑ Quality of life↑
Urrutia et al. (2020)	*Boxing:* 10-min warm-up session 30-min boxing training session, including combinations, heavy bag, and focus mitt drills 10-min cooldown period	90 min, 2×/week, 6 weeks (7–12 follow-up)	80–85% HR	Community	NR	HDS	Depression↑
Shearin et al. (2021)	*Boxing:* 10-min dynamic group warm up, education review on techniques and proper biomechanics 40-min training session were combined to include leg strength with punching combinations 10-min cool down focusing on trunk and lower extremity stretching session with patient education and group discussion	60 min, 2×/week, 12 weeks	RPE (4–7/10)	Community	80%	SSW, FW, DT (velocity, step length)	SSW: velocity↑ BW: velocity and step length↑ DT: velocity and step length↑
Combs et al. (2011)	*Boxing:* 20-min warm up: breathing and stretching exercises 45–60 min circuit training including functional and endurance and punching exercises, pushups, skipping, treadmill, cycling, running (3 min bouts with 1 min rests) 15–20 min cool-down focusing on breathing, stretching and strength	90 min, 2–3×/week, 12 weeks	Encouraging participants to train as intensely as they could tolerate	Community	100%	ABC, BBS, TUG, 6MWT, PDQL, UPDRS	Balance confidence↑ Balance↑ Mobility↑ Gait endurance↑ Quality of life↑ Disease severity↑
Dawson et al. (2020)	*RSB:* 30-min warm up: walking laps around the gym for 5 min, followed by 25 min of static and dynamic flexibility stretching of the entire body 45 min circuit training consisted of 12 rounds of various boxing routines using punching bags or a combination of circuit training and boxing. Sprints and jumping rope were performed for cardio. Agility exercises included footwork patterns that used agility ladders, steppers, and agility rings. Tennis balls and basketballs were incorporated into hand-eye coordination routines. Fine-motor and dexterity exercises utilized close pins and peg boards. Medicine balls, heavy battle ropes, and resistance and suspension bands were used for strength conditioning 15 min cool down with core exercises	90 min, 1×/week, 16 weeks	NR	Gym	96.7%	30s STS, TUG, EQ-5D	Lower extremity strength ↑ Mobility ↑ Quality of life ⟷
Hermanns et al. (2021)	*RSB:* Workouts include speed, agility, and endurance drills to improve hand-eye coordination, footwork, and general strength. Cognitive function drills and voice strengthening exercises are incorporated into each workout to promote dual-task performance and voice audibility respectively	60 min, 2×/week, 12 weeks	NR	Gym	NR	CES-D, PDQ-39	Depression↑ Quality of life↑
Moore et al. (2021)	*RSB:* 15-min warm up: focused on multi-planar active range of motion movements of the extremities and trunk 60 min circuit training focused on multi-planar active range of motion movements of the extremities and trunk. Movement patterns within the strength and endurance portion included bodyweight squats, punching a speed bag, and ball slams. This was followed by fine motor activities focused on improving upper extremity dexterity, such as tying shoes, picking up small objects from the ground, and stringing beads 15 min cool down with whole-body active range of motion movement patterns	90 min, 2–3×/week, 24 weeks	RPE (15–17/20)	Community	80%	FAB, TUG	Balance↑ Mobility↑
Horbinski et al. (2021)	*Boxing:* The program consists of hundreds of exercises/skill sets, broken down into three main phases. Phase one began with mastering a “set position” which established basic balance and holding a specific posture, with feet a little farther apart than shoulder width. In phase two, boxing footwork was practiced, wherein forward, side, and backward steps were made with increasing speed, based out of the set position and according to specific landmarks on the floor. The third phase involved mastering a series of punches, both in the air and at a bag, timed to maximize force based on proper balance, posture, and steps	2×/week	NR	NR	NR	15 s STS, 30 s CST	Lower extremity strength ↑ Balance ↑
Domingos et al. (2022)	*Boxing:* The program consisted of training punching movements and progressively adding in dual task challenges (i.e., going through a variety of punching combinations) *Traditional exercise:* The program received 10 sessions of group-based boxing training comparable to the previous group, but with added kicking techniques, weight shifting exercises and multi-directional stepping	60 min, 1×/week, 10 weeks	NR	Community	85%	Mini-BESTest, ABC, TUG, 6MWD, PDQ-39	Balance confidence↑ Balance↑ Mobility ⟷ Gait endurance↑ Quality of life↑
Sonne et al. (2021)	*RSB:* Workouts include aerobic training with walking laps around the gym or jumping ropes, resistance exercises with free weights and resistance bands, agility exercises including footwork patterns or large amplitude stepping, non-contact boxing with the use of punching gloves and punching bags, hand–eye coordination or manual dexterity activities, and stretching of the entire body	75–90 min, 5×/week, 12 mons	NR	NR	NR	30CST, FAB, TUG, 10MWT	Low extremity strength↑ Balance↑ Mobility↑ Gait velocity↑

RSB, Rock Steady Boxing; NR, no reported; ↑, Significantly positive effect; ↓, Significantly negative effect; ⟷, No effect; x/week, times of week; HR, heart rate; MDSUPDRS III, Movement Disorders Society Unified Parkinson’s Disease Rating Scale part III; UPDRS III, Unified Parkinson Disease Rating Scale; PDQ-39, Parkinson’s Disease Questionaire-39; HDRS, Hamilton Depression Rating Scale; PDQL, Parkinson’s Disease Quality of Life scale; TUG, timed up and go; dTUG, dual-task TUG; 6MWT, 6 min walk test; 6MWD, 6-min walk distance; BBS, Berg Balance Scale; ABC, Activity Specific Balance Confidence Scale; HDS, Hamilton Depression Scale; SSW, self-selected walking; FW, fast walking; BW, backwards walking; DT, dual task; EQ-5D, Brief quality-of-life survey the Euroquol-5D; STS, sit to stand; FAB, Fullerton Advanced Balance scale; CES-D, center for epidemiologic studies depression; Mini-BESTest, Mini-Balance Evaluation Systems Test; 10MWT, 10 Meter Walk Test; CST, Chair Stand.

### 3.5 Motor outcomes

#### 3.5.1 Lower extremity strength

Three studies (Dawson et al., 2020; Horbinski et al., 2021; Sonne et al., 2021) reported the effect of boxing on the lower extremity strength of patients, consistently indicating that boxing can effectively enhance their lower extremity strength. Sonne et al. (2021) observed a significant increase in the number of repetitions during the 30-s CST after 18 months of twice-weekly boxing exercise interventions. However, no significant improvement was observed at the 24-month mark compared to baseline data. Dawson et al. (2020) found that 16 weeks of weekly boxing training sessions, each lasting 90 min, resulted in a significant improvement in the number of repetitions during the 30-s STS test. Moreover, Horbinski et al. (2021) reported that twice-weekly boxing exercise effectively enhanced the performance in the 15-s STS test.

#### 3.5.2 Balance

Six studies (Combs et al., 2013; Combs et al., 2011; Domingos et al., 2022; Horbinski et al., 2021; Moore et al., 2021; Sonne et al., 2021) have reported the positive effects of boxing exercise on the balance abilities of patients, consistently noting varying degrees of improvement. Horbinski et al. (2021) found that participants who underwent twice-weekly boxing training significantly improved their performance in the 30-s single-leg stance test. Sonne et al. (2021) observed significant enhancements in the FAB Scale performance of patients at 6, 12, and 18 months after starting boxing interventions (no data were obtained at 24 months). Moore et al. (2021) also reported a significant increase in FAB Scale performance following a 24-week community boxing intervention for patients. Combs et al. (2011) demonstrated significant improvements in patients’ BBS and ABC scores during the 12 to 36-week intervention period, particularly among those with moderate to severe PD. When comparing 12 weeks of boxing exercise to traditional exercise therapy, Combs et al. (2013) found improvements in BBS and ABC performance in both groups, although only the traditional exercise group showed significant pre-to-post intervention differences, potentially due to specific balance training. Additionally, when comparing pure boxing exercise to boxing training combined with kicking techniques, both groups indicated significant improvements in Mini-BESTest and ABC performance after a 10-week intervention, with no significant differences between them (Domingos et al., 2022).

#### 3.5.3 Mobility

Six studies (Combs et al., 2013; Combs et al., 2011; Dawson et al., 2020; Domingos et al., 2022; Moore et al., 2021; Sonne et al., 2021) reported the effects of boxing exercise on the mobility of patients, consistently reporting significant improvements post-intervention. Combs et al. (2011) and Dawson et al. (2020) found that 12 weeks of thrice-weekly boxing training, as well as weekly boxing training over 16 weeks, significantly enhanced the TUG test performance in patients. Additionally, those who underwent 6 and 12 months of such training showed substantial improvements in TUG performance compared to their baseline assessments. Moore et al. (2021) corroborated these findings, noting that 24 weeks of biweekly to triweekly boxing training effectively improved TUG performance. However, no significant improvement was observed in the 18th and 24th months, as reported by Sonne et al. (2021). The investigation by Combs et al. (2013) further revealed that boxing significantly improved both TUG and dTUG performance compared to traditional exercise therapy over 12 weeks, but did not find a clear advantage in boxing. At the same time, 10 weeks of boxing training alone improved TUG performance in patients more effectively than boxing exercise combined with kicking techniques (Domingos et al., 2022).

#### 3.5.4 Gait

Six studies (Combs et al., 2013; Combs et al., 2011; Domingos et al., 2022; Sangarapillai et al., 2021; Shearin et al., 2021; Sonne et al., 2021) reported the effects of boxing on the gait parameters of patients, including gait velocity, gait endurance, stride length, stride width, and other sub-indices. Shearin et al. (2021) found that after 12 weeks of twice-weekly boxing training, patients showed significant improvements in self-selected gait velocity and step cadence, as well as backward walking velocity, gait velocity, step length, and gait variability index during dual-task conditions. Additionally, long-term intervention studies, such as Combs et al. (2011), have observed a significant increase in the walking distance on the 6MWT after 12 weeks of boxing exercise. For patients with mild PD, increased step cadence and stride length, along with decreased step width, were noted during GaitRite Walkway System tests at weeks 12, 24, and 36. Furthermore, the study results indicated that patients with moderate to severe disease required a longer training duration to achieve improvements in gait function compared to those with mild disease. Sonne’s study (Sonne et al., 2021) suggested that although there was no significant improvement in patients’ 10MWT time after 12 months of boxing training, there was an upward trend. Combs et al. (2013) research further demonstrated that 12 weeks of two to three times per week boxing training effectively improved patients’ 6MWT performance and gait velocity in the GaitRite Walkway System test compared to traditional exercise therapy. Research further demonstrated that 12 weeks of two to three times per week boxing training effectively improved patients’ 6MWT performance and gait velocity in the GaitRite Walkway System test compared to traditional exercise therapy (Sangarapillai et al., 2021). Domingos et al. (2022) compared the effects of boxing training alone versus boxing training combined with kicking techniques over 12 weeks. The results revealed no significant difference in the 6MWD between the two groups.

### 3.6 Non-motor outcomes

#### 3.6.1 Depression

Three studies (Hermanns et al., 2021; Patel et al., 2023; Urrutia et al., 2020) reported the effects of boxing on depressive symptoms in elderly patients, consistently finding that participation in boxing can improve these symptoms. Two studies (Hermanns et al., 2021; Patel et al., 2023) reported that participating in boxing exercises for 12 weeks, with sessions lasting 60 min and held twice per week, effectively improved scores on the HDRS. Additionally, research by Urrutia et al. (2020) showed that even a brief period of 6 weeks of high-intensity boxing training significantly improved HDRS scores. However, this improvement was not maintained at the 12-week follow-up assessment, reverting to baseline levels.

#### 3.6.2 Quality of life

Seven studies (Combs et al., 2013; Combs et al., 2011; Dawson et al., 2020; Domingos et al., 2022; Hermanns et al., 2021; Patel et al., 2023; Sangarapillai et al., 2021) reported the effects of boxing on the quality of life in patients. Six of these studies agreed that participation in such training can significantly improve patients’ quality of life (Combs et al., 2013; Combs et al., 2011; Domingos et al., 2022; Hermanns et al., 2021; Patel et al., 2023; Sangarapillai et al., 2021). Specifically, four studies (Combs et al., 2013; Combs et al., 2011; Hermanns et al., 2021; Patel et al., 2023) showed significant improvements in both PDQL and PDQ-39 scores following a 12-week program of boxing exercises, conducted two to three times per week for 60–90 min per session. Additionally, another two studies (Domingos et al., 2022; Sangarapillai et al., 2021) reported significant reductions in PDQ-39 scores with a shorter, 10-week training cycle involving one to three sessions of 60 min each per week. In contrast, research by Dawson et al. (2020) observed no significant change in EQ-5D scores after a 16-week course of weekly boxing exercise, each lasting 90 min.

#### 3.6.3 Disease severity

Four studies (Blacker et al., 2023; Combs et al., 2011; Patel et al., 2023; Sangarapillai et al., 2021) reported the effect of boxing exercise on the disease severity in patients. Most of these studies suggest that boxing exercises can effectively decelerate the progression of PD. Specifically, Patel et al. (2023) reported a significant reduction in the Movement Disorder Society-Sponsored Revision of the MDS-UPDRS III scores among elderly patients after 12 weeks of boxing training. Similarly, Blacker et al. (2023) observed a significant decrease in patients’ MDS-UPDRS III scores following 15 weeks of boxing training. A case series study noted that 12 weeks of boxing training significantly lowered the UPDRS scores of elderly patients, with this positive trend persisting during follow-up assessments at weeks 24 and 36 (Combs et al., 2011). However, Sangarapillai et al. (2021) found no significant improvement in the UPDRS scores of elderly patients after 10 weeks of boxing training compared to those who underwent sensory training, and symptom severity increased after a subsequent 10-week washout period.

### 3.7 Exercise safety and adherence outcomes

#### 3.7.1 Exercise safety and adherence

Seven studies (Blacker et al., 2023; Combs et al., 2011; Dawson et al., 2020; Domingos et al., 2022; Moore et al., 2021; Sangarapillai et al., 2021; Shearin et al., 2021) reported the effects of boxing on exercise safety and adherence in patients. Moore et al. (2021) and Shearin et al. (2021) reported an adherence rate of 80% among patients participating in boxing exercises. Dawson et al. (2020) reported a high participant adherence rate (96.7%) and retention rate (100%), with similar persistence (98%) and retention rates (100%) observed in studies by Sangarapillai et al. (2021) and Blacker et al. (2023). These findings collectively suggest that patients not only accept this intervention but also maintain participation over the long term. Combs et al. (2011) indicated high adherence, with all patients completing at least 24 training sessions within 12 weeks, and no adverse events occurred during the intervention, indicating high safety. Domingos’ (Domingos et al., 2022) study showed that 85% of participants completed the training course without any falls or other adverse events.

## 4 Discussion

The aim of this systematic review was to evaluate the effects of boxing exercise on lower extremity strength, balance, mobility, gait, depression, quality of life, disease severity, exercise safety and adherence in patients with PD. The primary findings of this systematic review suggest that long-term boxing exercise can effectively improve patients’ lower extremity strength (15-s and 30-s sit-to-stand, 30-s CST), balance (Functional Reach, Berg Balance Scale, Mini-BESTest, 30-s single-leg stance), mobility (TUG, dTUG), gait parameters (step length, stride width, 10MWT, and 6MWT), depression (CES-D, HDRS), quality of life (PDQ-39, PDQL, and EQ-5D), and disease severity (UPDRS). Additionally, adherence to boxing-based exercise was high among PD patients, and no adverse events were reported after long-term interventions. These findings provide the latest evidence supporting the positive effects of boxing on individuals with PD.

Research has shown that boxing exercises can significantly improve lower extremity strength, balance abilities, and self-confidence in balance control for patients with PD. The observed improvement in abilities may be attributed to the specific activities performed during each exercise session. Boxing, as an all-encompassing training method, incorporates aerobic, strength, and agility exercises, which challenge the participants’ lower extremity strength and balance ability (Combs et al., 2013; Combs et al., 2011; Sangarapillai et al., 2021). Strength is a vital component of physical fitness (Xu et al., 2023), and a strong correlation exists between lower extremity strength and balance ability. However, it is worth noting that only three out of 13 studies have assessed the impact of boxing on lower extremity strength in patients (Dawson et al., 2020; Horbinski et al., 2021; Sonne et al., 2021). Additionally, there is a paucity of literature on the effects of boxing exercises on the upper extremity strength in patients. Unlike traditional exercise programs that focus on static and dynamic balance drills, such as one-legged standing, boxing training enhances balance indirectly through activities integral to boxing techniques. These include dynamic balance maneuvers like reaching overhead while punching speed bags, as well as multidirectional reaching and stepping when following a trainer’s lead during focus mitt drills, both of which require participants to maintain and adjust their balance (Combs et al., 2011). Moreover, dual-task training, a key component of the boxing intervention, plays a significant role in improving balance (Domingos et al., 2022). Patients with PD undergoing physical movements simultaneously with high cognitive demand tasks experience heightened scrutiny of their balance control abilities. Studies indicate that these patients tend to exhibit diminished balance and gait function during dual-task scenarios (Kelly et al., 2012; Raffegeau et al., 2019). However, dual-task training has been found to effectively enhance their balance and gait performance (De Freitas et al., 2020; Strouwen et al., 2017). Consequently, the distinctive training approach of boxing not only improves the physical balance abilities of patients, but also strengthens their sense of self-efficacy when facing balance challenges. Additional research by Combs et al. suggested that patients with milder symptoms may show earlier improvements in balance compared to those with moderate to severe symptoms (Combs et al., 2013). The latter group may require a longer period of training to achieve the maximum benefits (Combs et al., 2011). This discrepancy could be due to the greater tolerance and capacity of mild PD patients to perform more repetitions within the boxing training framework (Combs et al., 2011).

Boxing exercise has been found to effectively enhance the mobility and gait functions of patients with PD, which are crucial for their quality of life (Curtze et al., 2016). As a multi-planar and full-body exercise, boxing requires the coordinated action of both upper and lower limbs within various footwork directions. The uniqueness of this training is its program diversity, such as variations in movement combinations, training complexity, e.g., coordinating upper and lower limbs in dual tasks, and dynamic adaptability, which involves adjustments according to the coach’s instructions (Shearin et al., 2021). As participants gradually master the techniques of boxing, the intensity of the exercise can be increased by enhancing punching speed and footwork power (Shearin et al., 2021). Additionally, as the training progresses, there is a corresponding increase in the complexity of movements, such as longer sequences and combinations of actions. Patients must also cognitively process instructions from the coach while performing movement tasks, such as advancing, retreating, and throwing punches. These elements challenge the patients’ dynamic neuromuscular control and improve their ability to complete various neuromechanical tasks, significantly improving their mobility and gait functions, including gait endurance, step length, stride width, and gait velocity. Studies suggest that enhanced motor control ability is fundamental for improved mobility, which requires the integration of visual, proprioceptive, and vestibular information (Miller Koop et al., 2019). High-intensity boxing may increase the quality and quantity of afferent information sent to cortical and subcortical areas, facilitating the processing and integration of sensory information in participants (Alberts et al., 2011). Imaging data also suggests that high-intensity exercise can lead to increased connectivity between subcortical and cortical structures, which may underlie the improvements in mobility and gait function (Alberts et al., 2016). However, research by Sangarapillai et al. (2021) and Domingos et al. (2022) found that after 10 weeks of boxing, patients did not show significant improvements in stride length, gait velocity, and mobility. This could be due to the shorter intervention period and lower frequency (10 weeks, 1–2 times per week), whereas previous studies had intervention periods of 12 weeks, 2–3 times per week (Combs et al., 2013; Combs et al., 2011; Moore et al., 2021; Sonne et al., 2021). It is noteworthy that some studies did not report medication use during the intervention period, which may obscure or exaggerate the effects of boxing training. To our knowledge, no studies have directly compared the effects of combined boxing training and medication therapy versus medication therapy alone or boxing training alone. However, research by Dibble et al. demonstrated that a 12-week combined intervention of exercise and medication therapy yielded superior improvements in muscle strength, UPDRS motor scores, and mobility in PD patients compared to resistance training or dopamine replacement therapy alone (Dibble et al., 2015). This suggests that the combined effects of exercise and medication therapy may be complementary. Currently, there is a lack of high-quality randomized controlled trials in this field, and future research is needed to validate the role of medication therapy in the outcomes of boxing training interventions.

Research has confirmed that boxing can effectively reduce depressive symptoms in patients with PD. The benefits of boxing are twofold: on one hand, it appears to alleviate depression by promoting the release of β-endorphin and increasing the availability of key neurotransmitters such as serotonin, dopamine, and norepinephrine, as well as by raising the levels of brain-derived neurotrophic factor (Basso and Suzuki, 2017; Fan et al., 2020). These physiological changes play a crucial role in alleviating depressive symptoms. Moreover, compared to repetitive exercises like walking or cycling on a stationary bike, boxing involves participants in complex movements and combinations that actively challenge cognitive functions, significantly enhancing their physical and psychological experiences (Patel et al., 2023). The social aspect of boxing is also significant. The camaraderie developed during training provides invaluable social support, which is vital for improving mood and reducing feelings of isolation (Hermanns et al., 2021). Physical activities, including boxing, have been shown to enhance self-esteem, self-evaluation, and a sense of accomplishment (Biddle and Asare, 2011; Wipfli et al., 2011), and are positively linked to self-efficacy (Wipfli et al., 2011). These factors may represent the underlying psychological mechanisms through which physical activities, including boxing, regulate depressive symptoms in patients. Notably, research indicates that just 6 weeks of high-intensity boxing training can significantly improve depressive symptoms in patients with PD.

The quality of life for patients with PD is primarily influenced by motor and non-motor symptoms, including balance, gait function, depression, and sleep disturbances (Balestrino and Martinez-Martin, 2017). Significant improvements in balance, gait function, and other motor abilities have been observed in participants after boxing interventions, contributing to an overall enhancement in quality of life. Enhancing quality of life is a major goal in public health research and practice (Fayers and Machin, 2015). As individuals age, their life satisfaction becomes linked to their health and mortality rates (Lim et al., 2017). Given that patients often struggle with gait disorders and cognitive decline, boxing, a physical activity that combines exercise with cognitive stimulation, could play a significant role in improving their overall quality of life. Studies have shown that participating in boxing can be a pleasurable experience for individuals, leading to high levels of satisfaction and a more positive attitude toward coping with the disease (Brunet et al., 2022; Hermanns et al., 2021). The enjoyable nature of boxing and the strong sense of community belonging it fosters may encourage a more positive attitude toward coping with the disease (Domingos et al., 2022). However, Dawson et al. (2020) reported that 16 weeks of boxing training, once a week for 90 min per session, did not significantly reduce patients’ EQ-5D scores. The choice of quality-of-life assessment tool is crucial. The EQ-5D is a widely used generic health status scale that assesses patients’ quality of life from five aspects: mobility, self-care, usual activities, pain/discomfort, and anxiety/depression. It is applicable to various diseases and patient groups. In contrast, the commonly used PDQ-39 specifically assesses the quality of life of patients by summarizing scores from 39 items, such as motor function, emotional state, and cognitive function, providing a comprehensive reflection of the patients’ quality of life status (Sangarapillai et al., 2021). This suggests that selecting the appropriate measurement tool significantly impacts the evaluation of intervention effects.

Scientific literature has suggested that boxing exercises significantly slow down the progression disease in PD of patients. This is likely due to the high-intensity nature of boxing, which promotes neural plasticity and thus delays disease progression (Frazzitta et al., 2014; Tollár et al., 2018). Boxing training typically involves circuit training, incorporating various components such as aerobic exercise, strength training, and footwork drills. Studies have showed that increased levels of brain-derived neurotrophic factor (BDNF) after high-intensity exercise are closely linked to improved motor function (Farrukh et al., 2023; Landers et al., 2019). It is hypothesized that boxing primarily stimulates the release of neurotrophic factors like BDNF, particularly since BDNF is essential for maintaining the survival and synthesis of dopaminergic neurons in the basal ganglia, thereby reducing disease severity (Combs et al., 2013; Morris et al., 2019). However, it is important to consider the research by Sangarapillai et al. (2021), which indicated that after 10 weeks of boxing training, participants did not show immediate significant improvements in disease severity compared to a sensory training group, with symptoms regressing during the subsequent 10-week washout period. This finding contradicts most studies that advocate boxing as a means to moderately improve disease severity and offer potential neuroprotective benefits (Blacker et al., 2023; Combs et al., 2011; Patel et al., 2023). The discrepancy in results may be due to the use of subjective scales by Sangarapillai et al. to monitor exercise intensity, potentially deviating from objective methods such as heart rate monitoring (Sangarapillai et al., 2021). This underscores the significance of exercise intensity on the symptom severity in patients and emphasizes the need for more precise methods of monitoring exercise intensity in future studies to clarify the specific impact of boxing on PD symptomatology.

The study revealed that the adherence rate for patients participating in a boxing exercise program was consistently above 80%, with no reports of adverse events. Notably, the reasons for patient dropout were unrelated to the boxing exercises themselves, but rather due to conflicts with their time and work commitments (Combs et al., 2011; Domingos et al., 2022). The primary reasons for dropout included scheduling conflicts with boxing training sessions (Patel et al., 2023), transportation difficulties (Shearin et al., 2021), family emergencies (Shearin et al., 2021), and other medical issues unrelated to PD (Combs et al., 2011; Patel et al., 2023; Shearin et al., 2021). Like other recommended forms of exercise, such as Tai Chi (Li et al., 2014; Vergara-Diaz et al., 2018) and dance (Volpe et al., 2013), these activities demonstrate high levels of adherence. Recently, attention has shifted to boxing exercises that are high dosage, sustained over the long term and are engaging, enjoyable and motivating for people living with PD (Blacker et al., 2023; Domingos et al., 2022; Patel et al., 2023; Sangarapillai et al., 2021; Shearin et al., 2021). One of the key advantages of boxing as an exercise modality is its community-based nature, which facilitates accessible and sustained exercise. The boxing program also includes a variety of therapeutic exercises, such as strength training, balance exercises, and agility drills, which not only enhance physical well-being but also foster social interaction, making it an engaging and sustainable form of exercise. In contrast, traditional treatments for PD patients are often confined to hospitals or home settings and typically involve short-term interventions lasting only 2–6 weeks (Carroll et al., 2017; Morris et al., 2019). These brief interventions may lead to higher dropout rates, as patients may struggle to maintain motivation and engagement over longer periods. Low adherence may lead to poor health outcomes and potential increased healthcare costs in people with PD. Therefore, boxing emerges as a highly compliant and safe physical rehabilitation strategy. It is recommended that this type of intervention be further promoted among more PD patients in the future to improve their quality of life and health condition. By integrating diverse exercise elements and fostering social interaction, boxing offers a comprehensive approach to enhancing the lives of PD patients.

The duration of boxing exercise interventions in various studies ranged from 10 to 96 weeks, with a frequency of 1–3 times per week and each session lasting 60–90 min. Five studies required patients to undergo high-intensity training programs (Combs et al., 2013; Combs et al., 2011; Moore et al., 2021; Sangarapillai et al., 2021; Urrutia et al., 2020), one reported moderate exercise intensity (Shearin et al., 2021), and another’s exercise intensity ranged from moderate to high (Blacker et al., 2023). Although current research cannot provide definitive conclusive information, the dosage of boxing exercise programs is consistent with the American Physical Therapy Association clinical practice guidelines for PD patients. According to these guidelines, patients should participate in aerobic exercise for 30–60 min, once or twice a week, or engage in moderate to high-intensity resistance training for 30–40 min per session, at least three times a week (Osborne et al., 2022). Regular physical activity reduces inflammation and oxidative stress, thereby protecting the nervous system (Hlebichuk et al., 2023). Studies have shown that high-intensity multimodal training provides additional benefits over low-intensity programs, including improved disease severity, stamina, and reduced fatigue (Landers et al., 2019). Moreover, high-intensity, high-frequency agility training significantly enhances depression symptoms, quality of life, and mobility performance in PD patients (Tollar et al., 2018). Notably, compared to moderate-intensity exercise, high-intensity exercise has a greater impact on regulating BDNF, resulting in increased post-training BDNF levels (Shulman et al., 2013). These findings are crucial for designing rehabilitation programs for neurodegenerative diseases since BDNF offers neuroprotection against brain degeneration, promotes the survival of dopaminergic neurons, and directly affects motor function (Rahmani et al., 2019). In conclusion, this systematic review reinforces the notion that high-intensity exercise interventions are a viable and effective rehabilitation approach for PD patients. Therefore, we advocate for high-intensity boxing exercise as an effective physical rehabilitation program for these patients.

However, the current systematic review has several limitations. Firstly, upon reviewing the included studies, we found significant diversity in the implemented methodology and design. Only three studies were RCTs, while the other 10 employed non-RCTs designs. This heterogeneity in study design and intervention characteristics—such as type and duration of exercise—introduces potential biases. Consequently, we refrained from calculating effect sizes, which somewhat limits the clinical significance of research findings. Secondly, there was notable variation in the study samples concerning disease stage, age, and gender. Additionally, many studies did not report the use of levodopa or other PD medications. Therefore, our results should be interpreted with caution, as they do not offer clear guidance on the dosage and mode of intervention based on participants’ age range and disease severity. Thirdly, although all studies used comprehensive, current, and validated assessment tools, inconsistency in outcome measurement tools was apparent. Lastly, our review was constrained to English-language articles, possibly missing research published in other languages. To conclusively determine the safety and efficacy of boxing exercises for improving motor and non-motor symptoms in PD patients, future research should include more homogenous PD patient populations and conducting RCTs. When designing exercise programs for elderly patients, it is essential to consider key factors such as individualized assessment, selection of exercise types, intensity and frequency of exercise, safety and supervision, and long-term adherence.

## 5 Conclusions and implications

Boxing exercises have demonstrated positive effects on both motor and non-motor symptoms in patients with PD, including improvements in lower extremity strength, balance, mobility, gait, depression, quality of life, and disease severity, particularly for patients with mild and moderate symptoms. This form of exercise is not only safe but also has high adherence, making it a valuable addition to medication and functional surgery. While evidence supporting the benefits of boxing exercises for PD patients continues to grow, there is still a need to establish the most effective treatment protocols. Future research on the effects of boxing exercise on PD patients requires scientific design, large sample size, long-term comprehensive intervention, and clear reporting standards.

## Data Availability

The original contributions presented in the study are included in the article/supplementary material, further inquiries can be directed to the corresponding author.
